# Effect of Temporal Relationships in Associative Rule Mining for Web Log Data

**DOI:** 10.1155/2014/813983

**Published:** 2014-01-23

**Authors:** Nazli Mohd Khairudin, Aida Mustapha, Mohd Hanif Ahmad

**Affiliations:** Faculty of Computer Sciences and Information Technology, Universiti Putra Malaysia (UPM), 43400 Serdang, Selangor, Malaysia

## Abstract

The advent of web-based applications and services has created such diverse and voluminous web log data stored in web servers, proxy servers, client machines, or organizational databases. This paper attempts to investigate the effect of temporal attribute in relational rule mining for web log data. We incorporated the characteristics of time in the rule mining process and analysed the effect of various temporal parameters. The rules generated from temporal relational rule mining are then compared against the rules generated from the classical rule mining approach such as the Apriori and FP-Growth algorithms. The results showed that by incorporating the temporal attribute via time, the number of rules generated is subsequently smaller but is comparable in terms of quality.

## 1. Introduction

The growth of web development technologies has led to the emergence of various web applications to suit human needs. Consequently, growing numbers of web-based applications and services produce huge amount of business and personal contents are stored in the web servers that are constructed in diverse formats. The resulting web log data are found to be lacking in structure and organization, hence making users feel disoriented and at loss during navigation due to information overload [1].

Web usage data is a valuable source for two avenues. One avenue is to exploit such data to provide effective browsing experience to users by detecting relevant information and creating some form of metaknowledge out of the available information. The second avenue is to improve the overall structure of the website content in such a way to facilitate searching and indexing of the web content. Overall, web usage data offers possibility to address the individual user needs and interests through their browsing and access patterns.

Web log usage mining falls under the umbrella of web mining, which focuses on discovering user navigation patterns from one web server to another. It also allows collection of information for the web pages, as the data collected will lead to the path of finding the web pages. The main objective of web log usage mining is to model, capture, and analyse the behavioural navigation patterns in effort to construct user profiles while they are interacting with the website [2]. Patterns that have been discovered by the mining techniques can be represented as collections of pages, objects, or resources that are frequently accessed by groups of users with common needs or interests [3].

Mining association rules from the web log data are able to provide web owners useful information regarding user behaviour and their navigation or browsing patterns. Such insights could later be used as input in developing dynamic and effective websites. In discovering navigation patterns from the web log data, existing work include the use of hypertext probabilistic grammar [4], graph theory [5], and formal methods [6] in order to model the user navigation sessions.

In predicting web page request to reduce access time and assist user navigation in a website, Shyu et al. [5] propose a method in finding user access pattern based on association rules and shortest path algorithm in graph theory. Subsequent works by Vasumathi and Govardhan [6] employ Formal-based Concept Analysis (FBCA) method to mine user interactions based on ordered lattice theory. The rules generated by FBCA are reported to be fewer than the set generated by a traditional Apriori but is comparable in terms of quality.

In association rules mining, the Apriori [7–9] and the FP-Growth [2, 8, 10] are two predominant algorithms widely used by researchers. The resulting association rules are able to aid the web owner to improve the structure or the content of the website. Work by Peng [2] in particular produces a topology of user best time access by combining the interest measure with the user profiles. Such topology is invaluable for improving the website organizational structures.

Nonetheless, the literature has shown very few works such as in Yu et al. [11] who captures temporal relationships from web log data. Lack of analysis on temporal relationships may result in loss of important time-series characteristics of the data, which will subsequently affect the quality of rules mined regarding the user navigation behaviours. As the application that relates to time-series characteristics such as online shopping histories, patient medical records, and banking nowadays are increasing, mining such data will give the web owner added advantage to enhance the content and structure of their websites. Because time is a virtually guaranteed dimension to be present in every data warehouse and virtually every data warehouse is a time-series [12], it is a great effort to incorporate this dimension in the mining process to discover interesting rules.

This paper will apply temporal-based approach to perform temporal relational rule mining for the web log data. The remaining of this paper will consist of Section 2 where we will discuss related works on temporal data mining, Section 3 will briefly describe the temporal-based mining approach, Section 4 will present the experiments on the discovery of temporal relational rules, Section 5 will discuss the effects of parameter used in the temporal-based approach, Section 6 will compare the results from temporal relational rule mining approach against the Apriori and FP-Growth, and finally Section 7 will conclude the paper with some indication for future works.

## 2. Related Work

Discovering association rules from a transaction records is basically finding patterns within the transactions. Most of the transaction records nowadays are captured with the time dimension making it virtually as a time-series transaction. The basic idea of temporal rule mining is to limit the search for frequent sets of items or itemsets only to the lifetimes of the itemsets member [12].

Temporal rule mining finds interesting patterns or rules in a large set of temporal data by incorporating the lifetime of the item within the associated time frame. Therefore, it may discover overlooked pattern or rules where temporal components have been ignored or only treated as numeric attribute [13]. Temporal rule mining focuses in time-series datasets that utilize the timestamps in the data. It has been used by researchers to discover knowledge from such datasets that can include temporal association rules [11–24], similar time sequences [25–27], as well as sequential patterns [28–32].

Seminal works in sequential patterns include Agrawal and Srikant [26] and Srikant and Agrawal [29]. In one of the more recent work, Laxman et al. [33] propose the use of temporal patterns which they call “generalized episodes” in order to facilitate in frequent episodes in synthetic and manufacturing status logs dataset. The study incorporates the duration constraints into the patterns definition. They have successfully developed the associated formalism and presented efficient algorithms for discovery of frequent generalized episodes in event sequences.

Time sequences discovery was first explored by Faloutsos et al. [25] and Agrawal and Srikant [26]. One recent work in mining similar time sequences is by Keogh et al. [27] who introduced the new problem of finding time-series discords. Time-series discords are described as subsequences of longer time-series that are maximally different to all the rest of the time-series subsequences. The study define time-series discords as a new primitive for time-series data mining by introducing algorithm called HOT SAX to efficiently find discords and demonstrate their utility on a host of domains.

Earlier study on the discovery of temporal association rules can be seen in Ale and Rossi [12] who incorporate time in performing association rule mining. They implement a temporal extension to the Apriori algorithm. Since then, various algorithms have been proposed to transform the data into temporal form. Li et al. [14] study the problem of time-related association mining by suggesting algorithm to extend the Apriori algorithm with effective pruning techniques. This research proposes calendar scheme as the framework for discovering temporal patterns. In the same year, Lee et al. [15] propose an algorithm called the Progressive-Partition-Miner to discover general temporal association rules in publication database. Subsequently, Verma and Vyas [17] also propose a new algorithm called the temporal *H*-mine that gives an efficient time sensitive approach for mining frequent item in the dataset using *H*-struct.

In 2006, Winarko and Roddick [13] introduce the Memory indexing for sequential pattern mining (MEMISP) in the discovery temporal rules from the frequent patterns, which resulted in a new algorithm named ARMADA. In 2007, Tseng et al. [18] implement a prediction model for user navigation called the Temporal *N*-Gram that is able to predict temporal navigation patterns. While Lee et al. [16] extract temporal interval relation data based on Allens theory [34], research by Gharib et al. [19] present incremental temporal association Mining (ITARM) that is able to reduce time requirement for generating new candidates by storing the candidate rules as 2-itemsets. Subsequently, Maragatham and Lakshmi [20] propose the utility-based temporal association mining (UTARM) by adopting support that are only relevant to the utility and time period. Next, based on the periodicity of data, Miao and Shen [21] improve the work by proposing mining periodic temporal association rules (MPTAR).

Incremental mining of general temporal association rules (IMTAR) is an algorithm that uses an extended TFP-tree to build the tree incrementally without the need to rescan the original database [22]. The evolution of temporal association mining techniques continues to grow with the introduction of general fuzzy temporal association rule mining algorithm (GFTARM) [23]. GFTARM solves the problem of mining fuzzy temporal association rules and Lal and Mahanti [24] examine the association rules mining in temporal database using the pipeline techniques.

Although many works have been done in temporal relational rule mining, very few works focuses on web log data. Publication database can be seen as preferred database used in many studies [15, 19, 22, 23]. Other researches use financial database [21] and customer database [16]. Temporal rule mining has only been recently explored in web log data by Yu et al. [11], hence, the basis of this research. Motivated by the current development of temporal association rule mining, this study attempts to apply the temporal-based approach with a time-based dataset and investigate the effect of parameter use in such approach towards a web log data and compare the number of rules discovered during the association rule mining with classic association rule algorithm.

## 3. Temporal Relational Rule Mining Approach

Assuming we have a transactional record of a supermarket for a period of one year. Goods sold in supermarket are not always available throughout the year due to certain circumstances such as goods only offered during sales, new products added in the middle of the year, or goods that have been sold are discontinued. For this reason, the support for each good will be different throughout different times in a particular year. Some goods have high support if the products are newly introduced, while other products may not have support for certain months, which will lead to product discontinuation. This situation may produce relationship that cannot be included as rules because of the support restriction [12]. Temporal relational rule mining approach caters this situation by incorporating time in the mining process, thus producing meaningful rules.

**Figure 1:**
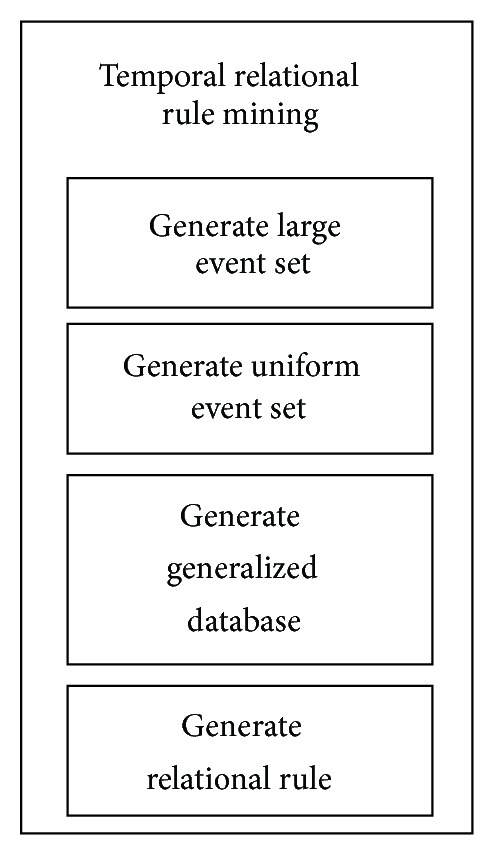
Steps in temporal relational rule mining.

In generating association rules for temporal relationship of the web log data, the database will be sorted accordingly to get the large event set in order to assess the frequency of each event. Then the events will be generalized into events with time interval. Association rules will be extracted out from these generalized events to discover the pattern of the web log data.  Figure 1 shows four general steps required to perform relational rule mining.

### 3.1. Generating Large Events Set

In temporal rule mining, a preprocessed dataset will be sorted by the user id (in web log case, user id will be the IP number) and transaction time. This will present all of the events for each user and event type can be identified. The first step after preprocessing is obtaining the large events set (LES), which consist of events that satisfies the user minimum support. The support for LES is decided by the proportion of user having the particular event type.

As in web log data, support is computed by the proportion of web user accessing times of the web pages. Discovering temporal interval relations is quite different from normal association rules as it needs to be done from the viewpoint of the set of customers entirely [16]. Normal association rules techniques will count the support of an item by the proportion of the transaction that has the item [35].

Prior to determining the LES from the web log dataset, the minimum support threshold for large event (MSLE) has to be specified. MSLE denotes the abstract level of the degree of generalization and is very important as the specified value will determine whether the output is a detailed or general event. Low MSLE will result in detailed events, while high MSLE will produce general events.

### 3.2. Generating Uniform Event Set

Once the large event set (LES) is obtained with the specified MSLE, events that are contained in the LES will be presented as a sequence of events. These sequence events will be sorted according to their access time, whereby a sequence of events represents a unit of temporal interval. In order to present the events with the temporal interval, a window size (WS) has to be defined. This is the maximum allowed time difference between the earliest and most recent time of event that occurred in the sequential pattern. Continuously occurred set of event is called uniform event set (UES). Even though events occur several times in several transactions within the window size, it will only be regarded as one.

The UES was obtained by first defining the minimum support threshold for uniform event set (MSUE). MSUE is the user specified value for frequency of any event occurrence within the interval. All event set that satisfies the MSUE will be considered in UES. Note that all UES are large event type, but large event type will not always be a uniform event type. This is because it is possible for events of large event type to occur heavily in one window size.

In generating UES, the web log dataset will be sorted according to individual users and their respective timestamps. Frequent event types will be calculated for every user while the nonfrequent event types from the events sequence will be deleted. Based on this set of frequent event types, a set of uniform event types and a set of sequences having that uniform event type for each user can be calculated.

**Figure 2:**
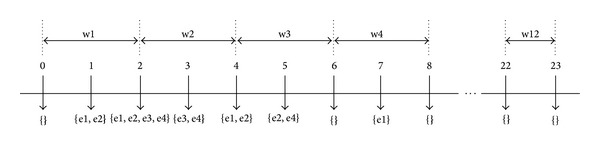
Tabulating large event type access time using temporal interval graph.

For example, if we have large event type of e1, e2, e3, and e4, their access time can be tabulated as shown in Figure 2. From this figure, assume that the 24 hours have been divided into 12 windows (w1 to w12). In w1 for instance, we can see one set of large event type consists of e1 and e2. This indicates that for the large event type e1 and e2 the access time for them is 1. The rest of the large event types are tabulated into the respective window size according to their access time.

### 3.3. Generating Generalized Database

Given the uniform event set, candidate temporal interval relation can be discovered by generating the generalized database (GD). Generalized database consists of generalized event that has continuously occurred within the interval of first access time, *V*_*S*_ and last access time, *V*_*E*_ for each user in the sequence event type. The sequence event types are part of the uniform event type set. In other words, GD will consist of *V*_*S*_ and *V*_*E*_ for every event type, which is the uniform event type for each individual user.

### 3.4. Generating Relational Rules

Once the generalized events with temporal interval have been obtained through GD, we will be able to specify a relation between any two intervals of the generalized events. In this paper we used the relations before (event *a* occurs before event *b*), meets (event *a* meets event *b*) and overlaps (event *a* starts within the time frame of event *b*), equals (event *a* starts and ends with event *b*), during (event *a* occurs during event *b*). By using these relations, a temporal relational rule that is based on the time constraint can be presented in the form of connections among the frequent temporal relations.

In temporal interval relational rules discovery, all candidate relational rules are discovered by comparing the interval of each two events types of each user. Lee et al. [16] has defined a temporal interval relation as *R*(*x*, *y*) = {*P*(*x*, *y*)∣(*x*, *y*) ∈ *Ω*, *P* ∈ IO} in which the temporal interval operator is IO = {before, equals, meets, overlaps, during}. By using the value of *V*_*S*_ and *V*_*E*_, the temporal interval relationship *P* is between *x* and *y*. *R*(*x*, *y*) are defined as

(1) before(x,y)≡  x·VE<y·VS,equals(x,y)≡  (x·VS=y·VS)∧(x·VE=y·VE),meets(x,y)≡x·VE=y·VS,overlaps(x,y)≡(x·VS<y·VS)∧(x·VE>y·VS),during(x,y)≡(x·VS>y·VS)∧(x·VE<y·VE).

Assume we have set of events with event type E1, E2 and E3 and their *V*_*S*_ and *V*_*E*_ values are presented as follows: {(E1, [1,2]), (E2, [2,3]), (E3, [1,3])}. We can present the temporal interval relation of this set of events as meets (E1, E2), during (E1, E3), and during (E2, E3).

After the generation of all candidate relation rules, support or frequency count for each of the rules will be calculated using the minimum support threshold for relation rule (MSRR) specified by user. MSR will determine the cut-off points for extracting the frequent relational rule and deleting the nonfrequent rules.

## 4. Experiments and Results

The objective of the experiments is twofold. The first is to transform a relational web log dataset into temporal relational dataset by incorporating the time characteristic. The effect of temporal parameters from large events (MSLE), uniform event set (MSUE), and relation rules (MSRR) are also investigated. After the temporal relational rules are mined, the second objective is to compare against the rules generated from classical rule mining approach using the Apriori and FP-Growth algorithms.

For the experiment, we used dataset that contains web log data from the Advanced Technical School in Novi Sad for one day, dated on November 16, 2009. The same dataset has also been used by Dimitrijević and Bošnjak [7] and Yu et al. [11] in their experiments. This dataset contains 5,999 transactions of web requests from various users.

Every line in the web log data consists of multiple information such as the IP address, time of request, requested URL, status code, size of response, referring URL, and web browser information. We follow Yu et al. [11] to select only IP address, time of request, and uniform resource locator (URL). For the purpose of relational rule mining experiment, the web log data has to undergo a series of data preprocessing steps before it could be transformed into a general temporal interval data.

### 4.1. Data Preprocessing

In this work, data preprocessing was carried out using a number of tools, which are WumPrep [7, 11], WebSpy Vantage [36], and Kirix Strata (http://www.kirix.com/). WumPrep is equipped with Perl script that has selection of filters to clean the data [37]. Unfortunately, the software is somehow outdated with no user interface, hence making it difficult to use. These entire tools are used throughout the data preprocessing activity. We started with WumPrep to remove irrelevant and automatic request. Then WebSpy is used to identify unique URL and getting the access time of the web pages. Kirix Strata is used as a tool to sorting and calculating support for events in the dataset.

Patel et al. [36] suggest Webspy Vantage to process and sort the data. We used the Webspy Vantage with web log data to generate unique URL visited by the users. From the results, we have identified 34 unique URL from the web log data. We also managed to get web pages visited by each user and recorded their first and last access time of the web pages. Webspy Vantage also provides a “sessionize” function, which will split the data according to the user-specified interval. Different from the “sessionize” filter in WumPrep, this function works with other aggregation functions such as to extract browsing time for better understanding of the results. Finally, Kirix Strata (http://www.kirix.com/) was also, explored and utilized to calculate the number of support for event throughout the mining process.

The raw dataset that we used for the experiment originally contains 5,999 lines of request. Data preprocessing includes removing the irrelevant as well as automatic requests. Details of the process are deliberated as follows.

- Remove irrelevant requests: irrelevant requests in a web log dataset contains such as icons, image, and any other resources that are embedded in the web page. Using the logFilter.pl script in the WumPrep we managed to discard 21.7% from the total lines of request leaving only 4,697. On top of that, we further removed 1,718 irrelevant requests that consists of status code other than 200, methods other than GET, and noisy data such as breaking loading page. We were then left with 2,975 lines of request.
- Remove automatic request: these types of request are most generated by spyware, crawlers, indexers, and bots. While the requests are captured by the web log, they actually do not represent actual request by the users. Hence, we treat this data as noise and should be removed. We first applied the *removeRobots.pl* script in the WumpPrep, but unfortunately no automatic requests were detected. We then had to perform manual scanning and manage to discard 848 lines of request leaving the cleaned data to 2,131 lines.

After preprocessing, the cleaned dataset now consists of 2,131 lines of request with the information of IP address, time of request of the web pages, and the requested URL.

### 4.2. Generating Large Event Set

In generating the large event set (LES), we defined the user session as one hour. We then extracted the candidate event type and calculated the supports for each of the event type. The support is determined by the number of visit times of every website by the user. We specified MSLE at 7%, which means the LES must contain event type that have at least been accessed by 7% of the users.  Table 1 shows the support count for each candidate event type sorted in descending order. Note that for every event type, an id has been assigned and will be used throughout the experiments.

From Table 1, event types 1 to 15 actually represent the unique URL in the web log data and were sequentially number based on the count of support for every event.

### 4.3. Generating Uniform Event Set

In obtaining the uniform event set, we used the sequence of event with temporal interval that is part of the LES. We defined the window size (WS) as 2, which returned us 12 windows within the 24-temporal interval. We then assigned every event into the timeline according to their access time. Event sequences that occurred several times in several requests within the same WS are summarized as one event within the temporal intervals. Frequency of each event was then calculated and candidates of uniform events were generated. To get the uniform event set, we defined the minimum support threshold for uniform events (MSUE) as 8%.  Table 2 shows the uniform event set that was extracted, where the support for each of the event type of uniform event set is calculated. The event types in this table are those that satisfied the specified MSUE.

### 4.4. Generating Generalized Database

In order to discover candidate temporal interval, a generalized database (GD) was created. Generalized database consists of generalized events with records of time of first access, *V*_*S*_ and time of last access, *V*_*E*_ for each user in the sequences of event type. Note that every event for each user originated from the uniform event set. Extract of the GD is shown in Table 3.

As shown in Table 3, for every user (user is distinct by the IP address), all the event sequences that are included in the uniform event set are tabulated with their *V*_*S*_ and *V*_*E*_.

### 4.5. Generating Relational Rules

**Figure 3:**
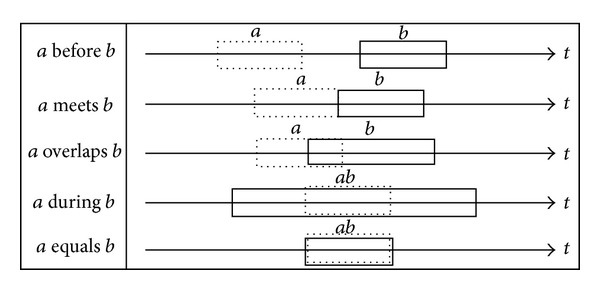
Type of relation between events.

From the generalized database (GD), relations between two intervals of the events are then extracted. The types of relations that were considered in this experiment are summarized in Figure 3.

Based on Figure 3, *a* and *b* represent the events, while *t* is the timeline where the events occurred. For instance, *a* before *b* represents an event that occurred before the next event. The candidate relation rules were generated by comparing the time interval between two events, *a* and *b*, for each user. We then calculated the support for each candidate relation rule. In addition, we discarded candidates that have no relation, which happened when a user accessed only to one website at a particular interval with no subsequent access. We then defined the minimum support threshold for relational rule (MSRR) as 5%, which will extract only the relation rules that satisfied this threshold and discard all the infrequent relation rules. Rules extracted from the generalized database are shown in Table 4.

As shown in Table 4, we have extracted 22 temporal relational rules from the temporal relational rule mining experiment. One example of temporal rule extracted is the rule 2 (before) 3 with support of 20.7%. This rule implies that 20.7% of the users who visit website 3 (http://www.vtsns.edu.rs/ispit_raspored_akt.php) will visit website 2 (http://www.vtsns.edu.rs/ispiti.php) first. This discovery may be able to help the webmaster to organize their contents according to the user behaviors such as putting appropriate hyperlinks for frequent interconnected page. Note that, however, the websites are part of the existing dataset from Dimitrijević and Bošnjak [7], hence, may not be necessarily accessible at present.

## 5. Effect of Parameter Use in Temporal Relational Rule Mining

Temporal relational rules mining requires user to specify a number of parameters throughout the mining process. While well-known association rule mining algorithms such as Apriori and FP-Growth use minimum support threshold in the generation of rules from a dataset, temporal relationship use the minimum support threshold for large events (MSLE) to generate the large event set, minimum support threshold for uniform event set (MSUE) to generate the uniform event set, and finally minimum support threshold for relation rule (MSRR) to generate the frequent relational rules. We use the same dataset to analyse the effect of those parameters used in the mining task.

### 5.1. Effect of MSLE

**Figure 4:**
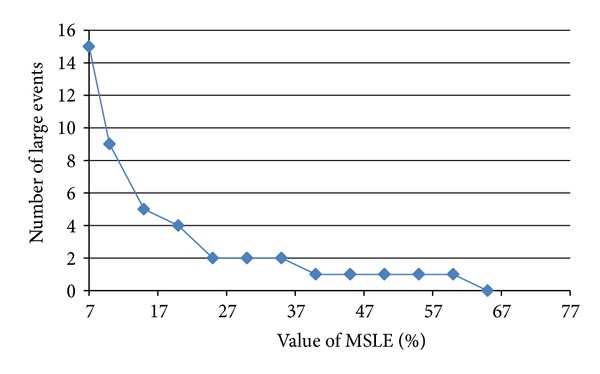
Effect of MSLE parameters.

MSLE is used to find large event sets, which represent frequent events in the dataset. MSLE is important to eliminate infrequent events so as to reduce search space and computational time, while maintaining the accuracy of the mining process. In this part of experiments, we analysed the number of large events set whenever the MSLE values changed. As shown in Figure 4, the smaller the number of MSLE, the higher number of large event set will be generated. In case where the choice of MSLE value is uncertain, we could choose the value when the number of large events generated is static or changed very little. Figure 4 shows the changes in number of large events with various MSLE values.

From this figure, we can see that the number of large events generated was highest when smaller MSLE been defined and getting reduced when the MSLE is higher.

### 5.2. Effect of MSUE

**Figure 5:**
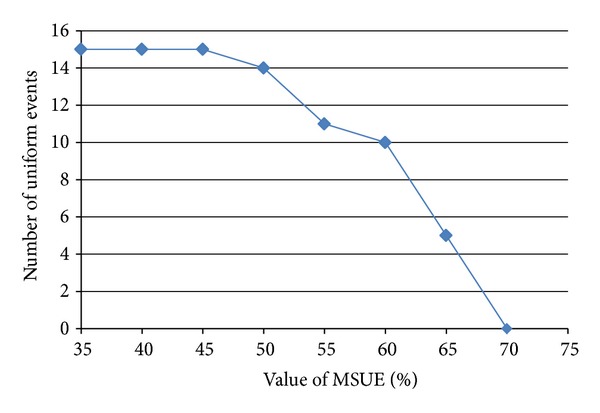
Effect of MSUE parameters.

Generation of the uniform event set depends on the minimum support threshold for uniform event (MSUE) value, which represents any occurrence of events within the intervals from the large event set. As events should occur continuously, the decision to obtain this uniform event set will discard any event that resulted from repeated access by the same user in a short period of time. In this analysis, we compared the number of uniform events against the changes of MSUE value. From Figure 5, we can see that MSUE will generate a more uniformed UES whenever the value of MSUE is greater. In the case where the choice of MSUE value is uncertain, we could take the value where the number of large events starts to become static or changed very little.

### 5.3. Effect of MSRR

**Figure 6:**
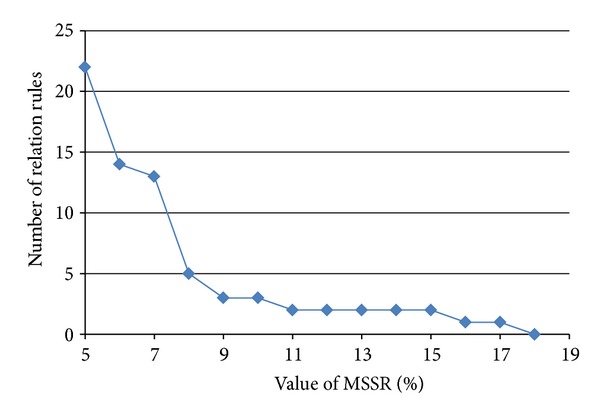
Effect of MSRR parameters.

The role of minimum support threshold for relation rule (MSRR) is to determine if the candidate relation rules are frequent in order to obtain only the set of interesting relation rules. MSRR will discard infrequent rules that do not satisfy the threshold. To analyse its effect, we compared 268 candidate relations with the various value of MSRR as shown in Figure 6.

Based on Figure 6, the results showed that the greater the MSRR, the more the number of relational rules become reduced.

## 6. Comparative Experiments

The Apriori and FP-Growth algorithms are two of the most widely known and used in association rule mining. The fundamental principle in Apriori for generating the rule is that if an itemset is frequent, so are the subsets. Hence, the support for an itemset will never exceed the number of support for it subsets. On the other hand, FP-Growth allows the discovery of frequent itemsets without any generation of candidate itemset. This is carried out by building a compact data structure known as the FP-Tree, from which all the frequent itemsets will be extracted. Temporal relation rule mining approach, however, is different from these two-known algorithms due to consideration of time interval in the mining process. Meanwhile, the Apriori and FP-Growth algorithms discover interesting patterns and association rules without considering the time of transactions.

In order to investigate the effect of time in association rule mining, we experimented the same dataset [7, 11] used in the temporal relational rule mining with Apriori and FP-Growth algorithms. The objective of this experiment is to see the difference in term of number of rules generated without the influence of time intervals. Following [7, 11], the experiments were carried out using Weka to run both algorithms. We also used the same value of support threshold as MSLE, which is 7% for both algorithms.

**Figure 7:**
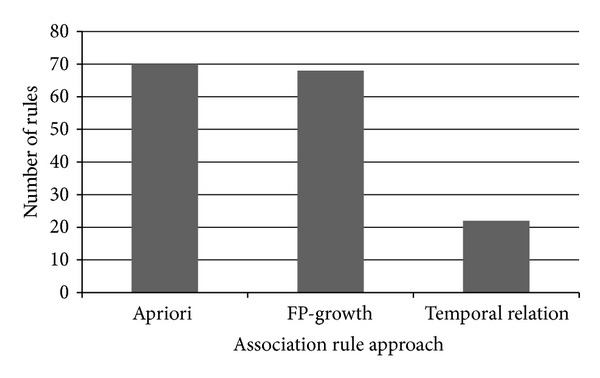
Number of rules generated by different association rule approach.

Dimitrijević and Bošnjak [7] propose to use pruning in order to reduce the number of interesting relation rules generated by an Apriori algorithm. However, in this experiment, the pruning scheme was not incorporated because there are no strong rules due to strong connectedness of the web pages detected in the generated rules. The results from the experiment are shown in Figure 7.

From Figure 7, we can see that by incorporating time interval in the temporal relation approach, the number of generated rules is significantly reduced. This means without the time factor, rules generated will be based on the frequent events of user entering any website in the dataset. Assume that a user repeatedly accessed a website (i.e., five visits) in a short time. Both Apriori and FP-Growth algorithms will count the frequency as five. However, such visit will be tabulated within the same window interval in temporal relational rule mining. Therefore, even if the visits occurred several times during several transactions in that particular window size, it will be summarized into only one event.

As most of datasets such as web logs, retail transactions, or medical histories include time as one of their dimensions; temporal relational rule mining has the advantage to uncover knowledge that could be potentially missed with regard to time. For example in the retail industry, mining temporal relational rules for product transactions throughout some specific time period would reveal products that do not exist at some point within the entire duration. According to Ale and Rossi [12], temporal association rules mining has made possible to eliminate outdated rules based on the criteria defined by the users. It is also possible to remove itemsets that are already obsolete based on their time interval, thus reducing the effort to determine the frequent rules. Ultimately, this means we are able to minimize the search space, hence, the computational cost.

Another important issue in rule mining is setting the parameter for frequency support count that has be satisfied in order to generate interesting rules. Temporal relation rule approach uses several parameters, which hold threshold values, of MSLE, MSUE, and MSRR along the process of rule mining. In the Apriori and FP-Growth, there is only one threshold value needed, which is the minimum support to generate the rules. The analysis of MSLE, MSUE, and MSRR used in temporal relational rule approach has shown that for different value of the parameters, we can expect significant impact to the variables involved. Comparative experiments between temporal relational rule mining and Apriori/FP-Growth algorithms also showed a substantial reduction in the number of generated rules by temporal relational rule mining.

## 7. Conclusion

In this paper, we have presented in detail the mechanism to model temporal relationships from the web log data. Temporal relational rule mining approach consists of several essential steps beginning from generating the large events set from the cleaned dataset, generating the uniform event set from the large event set, generating a generalized database to obtain candidate temporal intervals, and finally generating temporal relational rules from the generalized database.

We have experimented with web log data using temporal relational rule mining approach to discover interesting relational rules. The approach that incorporates time interval into the mining process has significantly reduced the number of generated rules as compared to other association rule mining algorithms such as the Apriori and FP-Growth. In addition, we also analysed the effect of parameters used in the temporal relational rule approach, which include MSLE, MSUE, and MSRR. In the mining process, the definition of these parameters should clearly reflect the organization needs. These parameters will have direct influence on the number of interesting rules generated to help the organization to discover any hidden information or knowledge from their web log data.

Based on this experience, we found that there is no standard or reference tool for treating the web log data. Preprocessing web log data is more tedious due to several reasons. For example, the URL has to be represented in the form of id to be used throughout the mining process. Modelling the temporal relationship in the web log data is another challenge because in most cases, setting of parameters such as MSLE, MSUE, and MSRR is intuitive and has to be defined specifically to reflect the organization's need. In the future, we hope to develop a tool to cater temporal aspects from web log dataset during preprocessing. As most data warehouses record time as one of their dimension, the development of such tool may benefit organizations in term of providing better websites to the users.

## Figures and Tables

**Table 1:** Large event set.

Event type	Support
1	133
2	86
3	63
4	56
5	38
6	26
7	26
8	26
9	22
10	21
11	19
12	19
13	18
14	18
15	16

**Table 2:** Uniform event set.

Event type	Support
1	10
2	10
3	10
6	10
14	10
12	9
9	9
8	9
5	9
4	9
15	9
10	8
11	8
13	8
7	7

**Table 3:** Generalized database.

IP address	*V*_*S*_	*V*_*E*_	Event type
147.91.173.31	0:02:28	0:02:51	6
147.91.173.31	0:02:52	0:03:06	9
147.91.173.31	0:03:07	9:19:10	1
147.91.173.31	0:03:13	8:45:44	4
147.91.173.31	0:03:41	0:04:19	7
147.91.173.31	0:04:20	0:04:20	8

77.239.68.36	0:08:54	1:12:53	1
77.239.68.36	0:08:57	1:12:55	2
77.239.68.36	0:09:00	0:09:01	3

**Table 4:** Relational rules.

Relational rules	Support (%)
2 [before] 3	20.7
2 [before] 5	14.5
1 [before] b2	11.7
1 [before] b3	11.7
1 [before] b4	11.7
3 [before] d2	11.0
1 [before] 5	9.7
2 [before] 1	9.7
2 [before] 4	9.7
3 [before] 5	9.7
2 [meets] 3	9.0
2 [during] 1	7.6
5 [during] 2	7.6
3 [during] 1	6.2
10 [before] 1	5.5
12 [during] 8	5.5
3 [before] 1	5.5
4 [before] 14	5.5
4 [before] 5	5.5
5 [before] 1	5.5
6 [before] 1	5.5
8 [before] 1	5.5
